# Analysis of *ANK3* and *CACNA1C* variants identified in bipolar disorder whole genome sequence data

**DOI:** 10.1111/bdi.12203

**Published:** 2014-04-10

**Authors:** Alessia Fiorentino, Niamh Louise O'Brien, Devin Paul Locke, Andrew McQuillin, Alexandra Jarram, Adebayo Anjorin, Radhika Kandaswamy, David Curtis, Robert Alan Blizard, Hugh Malcolm Douglas Gurling

**Affiliations:** a Molecular Psychiatry Laboratory, Division of Psychiatry, University College LondonLondon, UK; b Knome Inc.Cambridge, MA, USA; c Department of Psychological Medicine, Queen Mary University of LondonLondon, UK

**Keywords:** allelic association study, ankyrin 3, bipolar disorder, DNA sequencing, genetic, L-type calcium channel

## Abstract

**Objectives:**

Genetic markers in the genes encoding ankyrin 3 (*ANK3*) and the α-calcium channel subunit (*CACNA1C*) are associated with bipolar disorder (BP). The associated variants in the *CACNA1C* gene are mainly within intron 3 of the gene. *ANK3* BP-associated variants are in two distinct clusters at the ends of the gene, indicating disease allele heterogeneity.

**Methods:**

In order to screen both coding and non-coding regions to identify potential aetiological variants, we used whole-genome sequencing in 99 BP cases. Variants with markedly different allele frequencies in the BP samples and the 1,000 genomes project European data were genotyped in 1,510 BP cases and 1,095 controls.

**Results:**

We found that the *CACNA1C* intron 3 variant, rs79398153, potentially affecting an ENCyclopedia of DNA Elements (ENCODE)-defined region, showed an association with BP (p = 0.015). We also found the *ANK3* BP-associated variant rs139972937, responsible for an asparagine to serine change (p = 0.042). However, a previous study had not found support for an association between rs139972937 and BP. The variants at *ANK3* and *CACNA1C* previously known to be associated with BP were not in linkage disequilibrium with either of the two variants that we identified and these are therefore independent of the previous haplotypes implicated by genome-wide association.

**Conclusions:**

Sequencing in additional BP samples is needed to find the molecular pathology that explains the previous association findings. If changes similar to those we have found can be shown to have an effect on the expression and function of *ANK3* and *CACNA1C*, they might help to explain the so-called ‘missing heritability’ of BP.

Bipolar disorder (BP) is a common disease with a worldwide average population prevalence of 1.4%, which rises to 2.4% if bipolar spectrum disorders are included 1. Twin and family studies indicate that BP is genetically related to some types of unipolar affective disorder 2. The genetic heritability of BP is thought to be between 79% and 93% 3–6, with a ten-fold increase in risk to the relatives of probands with BP 7. Many linkage studies of specific chromosomal regions and whole genomes in multiply affected families support the presence of locus heterogeneity, with multiple susceptibility loci 8–10. About half of the segregation analyses of systematically ascertained families imply that BP has an autosomal dominant mode of inheritance 11. Other models have favoured a single major locus with a polygenic multifactorial background and pure polygenic transmission. Linkage and linkage disequilibrium (LD) analyses demonstrate locus heterogeneity 12,13. Genome-wide association studies (GWAS), meta-analyses, and replication studies focusing on BP have been carried out on combined cohort sizes of up to 7,481 cases and 9,250 controls 14–17. These and other single-locus case–control association studies have repeatedly implicated the L-type calcium channel α1C subunit (*CACNA1C*) and ankyrin 3 (*ANK3*) genes in BP. The strongest allelic association signal in *CACNA1C* is localized entirely within intron 3 of the gene with the single nucleotide polymorphisms (SNPs) rs1006737 (p = 7.0 ×10^−8^) 14,18,19, rs4765913 (p = 1.52 × 10^−8^) 14, rs4765914 (p = 1.52 × 10^−8^) 20, and rs1024582 (p *=* 1.7 × 10^−7^) 17,21. GWAS results across five different psychiatric illnesses further implicate rs1024582 in susceptibility to both BP and schizophrenia, assuming that there has not been substantial misdiagnosis, especially where schizoaffective BP cases are included in the schizophrenia group 20. Intron 3 of *CACNA1C* contains a chromosomal region with high levels of LD, strong mammalian conservation, and multiple sites designated by the ENCyclopedia of DNA Elements (ENCODE) project as being able to affect gene expression. Studies show that that the presence of the rs1006737 *CACNA1C* BP risk variant may have an impact on certain brain activities. One study showed that the rs1006737 risk variant in healthy males is associated with lower extraversion, trait anxiety, paranoid ideation, and higher harm avoidance 22. The rs1006737 risk variant has been associated with increased amygdala functioning observed by magnetic resonance imaging during emotional processing; the enhancement of activation leads to impaired facial emotion recognition in BP patients 20,23–26. There has been conflicting evidence as to whether the presence of the *CACNA1C* variant results in brain volumetric alteration. Some reports state that this SNP has been associated with brainstem alterations, increased grey matter density, as well as a cortical volume increase 27–29. A conflicting study did not report any association between this SNP and brain volumetric alterations 30. Mutations/variants located in intronic regions can also affect the stability of RNA and protein expression, and can have a strong effect on the transcriptional regulation of the gene.

In *ANK3*, the strongest evidence for allelic association comes from SNPs rs10994338 (p = 1.20 ×10^−7^) 31, rs4948418 (p = 8.93 × 10^−9^) 15, rs10994336 (p = 9.1 × 10^−9^) 14,26,32–34, rs10994397 (p = 7.1 × 10^−9^) 17 at the 5′ end, and the SNP rs9804190 (p = 1.20 × 10^−4^) 17,26,35 at the 3′ end of the longest isoform (NM_001204403) of the gene. These regions are over 340 kb apart and appear to be independently associated with BP, with no significant interactions between SNPs from the two regions 32. However, the existing data on *ANK3* show that only low-frequency aetiological base-pair (bp) changes are present with an odds ratio less than 1.35 for BP 17. The SNP associations are not replicated in every study 36–39; however, the *ANK3* association has been reported in several different ancestral populations 36,40–42. Several novel, rare potential aetiological bp changes have been identified by us through sequencing the gene in our samples. These were selected for having haplotypes associated with BP 43. Doyle et al. 44 sequenced the 8 kb brain-expressed exon 48 of *ANK3* but could not find potential aetiological bp changes that were associated with BP. This exon is of recent evolutionary history, and variation in the exon appears to be tolerated. Sequencing analysis of *ANK3* demonstrated the impact of heterogeneity on replication of allelic associations, even within well-defined ancestral populations 43. mRNA analysis has detected differential regulation of distinct *ANK3* transcription start sites and coupling of specific 5′ ends with 3′ mRNA splicing events, suggesting that brain-specific *cis*-regulatory transcriptional changes might be relevant to BP molecular pathology 45. Gene network analysis and test of epistasis have found further support for an association of *ANK3* with BP 46,47. The genetic variants associated with disease have no known biological function. However, one study showed that the presence of the rs10994336 BP risk variant in healthy males might predict lower novelty seeking, lower behavioural activation scores, and high startle reactivity 22. In healthy volunteers, rs10994336 may be associated with reduced white matter integrity in the anterior limb of the internal capsule, as well as with altered set-shifting and decision-making 48. These findings may be consistent with previous diffusion tensor imaging studies in patients with BP 49–54 and core phenotypes of BP 55–58. Lithium has been shown to alter *Ank3* mRNA levels in the mouse brain 59, and lithium and sodium valproate have been shown to change *Ank3* protein amounts in rat neuronal dendritic spines 60. In another animal model, RNA interference of *Ank3* in the hippocampus dentate gyrus induced a reduction of anxiety-related behaviours and increased activity during the light phase, which were attenuated by chronic treatment with the mood stabilizer, lithium. Similar behavioural alterations of reduced anxiety and increased motivation for reward were also exhibited by *Ank3*+/− heterozygous mice compared with wild-type *Ank3*+/+ mice 61.

Given the typical natural history of BP, which consists of episodes of both mania and depression with complete recovery very often between episodes, it can be argued that genetic susceptibility will involve aetiological bp changes influencing the control of gene expression and mRNA translation rather than mutations creating structural protein abnormalities. Therefore, we chose whole-genome sequencing (WGS) rather than exome sequencing in order to be able to investigate intronic and non-coding control regions of susceptibility genes along with the exonic coding regions.

## Materials and methods

### Subjects

This study included 1,510 affected research subjects with BP. These were sampled in three cohorts. The first cohort, UCL1, included 506 research subjects with bipolar I disorder (BP-I), defined by the presence of mania and hospitalization according to Research Diagnostic Criteria (RDC) 62. UCL1 was included in the previously reported mega-analysis by the Psychiatric Genetic Consortium (PGC) BP GWAS 17. The second and third cohorts, UCL2 and UCL3, consisted, respectively, of 593 and 411 subjects with BP-I or bipolar II disorder (BP-II). Ancestry screening was used as a selection criterion for the inclusion of cases. Samples were included if at least three out of four grandparents were English, Irish, Scottish, or Welsh and if the fourth grandparent was non-Jewish European, before the European Union enlargement in 2004. The sample of 1,095 controls comprised 614 screened subjects who had no first-degree family or personal history of psychiatric illness and an additional 481 unscreened normal British subjects, obtained from the European Collection of Animal Cell Cultures (ECACC). National Health Service (NHS) multicentre research ethics approval was obtained. All participants provided signed consent.

Research subjects with BP had been given an NHS clinical diagnosis of ICD-10 BP and then needed to fulfil RDC 62 for BP with clinical data collected by the lifetime version of the Schizophrenia and Affective Disorder Schedule (SADS-L) 63. DNA samples were collected from blood samples from the UCL1 cohort, saliva samples for the UCL2 cohort, and a mixture of both blood and saliva for the UCL3 samples. DNA from blood samples was extracted using a standard phenol–chloroform method and from saliva samples using the Oragene protocol for DNA extraction (DNA Genotek, Ottawa, ON, Canada).

### WGS

WGS was performed on 99 of the subjects with BP-I selected from all our cohorts who had a positive family history of BP or bipolar spectrum disorder and an early age at onset. The genomic DNA was sequenced using 100 bp paired-end reads on a Hi-Seq 1000 (Illumina Inc., San Diego, CA, USA). Sequence data alignment to the National Center for Biotechnology Information human reference genome 37.1 (hg19) and variant calling was performed using the CASAVA 1.8.2 pipeline at Illumina (http://res.illumina.com/documents/products/technotes/technote_snp_caller_sequencing.pdf). The sequence data from these individuals were further analysed and annotated using kGAP (Knome Inc., Boston, MA, USA).

### Variant selection

*ANK3* and *CACNA1C* non-synonymous variants present in the coding exons were identified using the Knome VARIANTS software (Knome Inc.) (*Supplementary Table 1*). The same software was used to identify variants in the 5′ untranslated region (UTR), 3′UTR, splicing sites [donor site consists of 5 bp in the exon and 6 bp in the intron, acceptor site consist of 3 bp in the exon and 20 bp in the intron 64], promoter region (1,000 bp from the first exon of every coding isoform), and the third intron of *CACNA1C* (*Supplementary Table 1, Supplementary Fig. 1, Supplementary Fig. 2*). Allele counts for each SNP in the BP samples were compared to those from the 372 European samples in the 1,000 Genomes (1,000G) Project (phase 1, version 3; http://ftp://ftp.1000genomes.ebi.ac.uk/vol1/ftp/release/20110521/ALL.wgs.phase1_release_v3.20101123.snps_indels_sv.sites.vcf.gz). SNPs for which the variant allele was more common in subjects with BP than in the 1,000G Project data, significant at p < 0.05 using Fisher's exact test, were chosen for genotyping in the complete UCL case–control sample. Variants which were present in poly-base regions and insertions in repeat regions were excluded from genotyping.

The variants in the third intron of *CACNA1C* were selected if they met four criteria:

- Located in the third intron of *CACNA1C* between flanking high recombination peaks (chr12:2271532-2425994 hg19);
- Located in a putative functional site defined by being an ENCODE-marked element (65);
- Located in a conserved non-coding sequence, determined using the mammalian conservation track on the University of California Santa Cruz (UCSC) genome browser (http://genome.ucsc.edu/) or by their Genomic Evolutionary Rate Profiling (GERP) scores;
- Not in a repeat region.

Each variant was validated by confirming the bp call confidence using individual Binary Alignment/Map (BAM) files before genotyping. Bioinformatic analysis to determine potentially functional SNPs was carried out using the UCSC genome browser, Alibaba2.1 (http://www.gene-regulation.com/pub/programs/alibaba2/index.html), targetscan 6.2 (http://www.targetscan.org/), miRanda (http://www.microrna.org/microrna/home.do), PolyPhen-2 (http://genetics.bwh.harvard.edu/pph2/), and Sorting Intolerant From Tolerant (SIFT) (http://sift.jcvi.org/).

### Genotyping

Genotyping for the selected SNPs in 1,510 BP cases (UCL1, UCL2, and UCL3 samples) and 1,095 ancestrally matched controls was performed in-house with allele-specific polymerase chain reaction (PCR) using KASPar reagents (KBiosciences, Hoddesdon, UK) on a LightCycler 480 (Roche, Burgess Hill, UK) real-time PCR machine. For all SNPs genotyped, 17% of samples were duplicated to detect error and confirm the reproducibility of genotypes. Allele-specific primers were designed for each of the SNPs using Primer Picker (KBiosciences), as shown in *Supplementary Table 2*. All these data were analysed to confirm Hardy–Weinberg equilibrium (HWE). Allelic associations for SNPs were performed using Fisher's Exact test. Significance values shown for all analyses are uncorrected for multiple testing, and a cutoff significance value of p < 0.05 was used.

### Burden analysis

A burden analysis was performed on the data separately for *ANK3* and *CACNA1C*. A chi-square test was used to compare the numbers of case and control individuals carrying one or more of the variant alleles against the numbers of case and control individuals who were found to be homozygous for the reference alleles at all of the loci tested.

### Haplotype analysis

Haplotype analysis was performed using Haploview 66 to determine the LD between GWAS-associated SNPs and the rare variants reported here. Haplotype blocks were determined using a solid spine of LD (D′ = 1).

## Results

### Variant calling and selection

WGS in 99 samples with BP produced a mean depth coverage of 37.0 with 90% of the genome sequenced. A total of 0.12% of bases were heterozygous and the transition/transversion (Ti/Tv) ratio was 2.0 (see *Supplementary Table 3*).

Using the criteria set out above, 82 *ANK3* and 43 *CACNA1C* variants were identified in the coding exons, 5′UTR, 3′UTR, splicing sites, and promoter region; and a further 108 *CACNA1C* variants were identified in the intron 3 region 12:2271532-2425994 (*Supplementary Table 1, Supplementary Fig. 1, Supplementary Fig. 2*).

These variants were further filtered using a cutoff threshold of p < 0.05 using Fisher's exact test against the 1,000G Project 67 European allele frequencies. After filtering, three variants remained in *ANK3* and these comprised two known SNPs, rs184389434 and rs139972937, and a previously unreported SNP at position ss825679002; ten *CACNA1C* variants remained after filtering and these comprised five known SNPs (rs146482058, rs79398153, rs191953785, rs112312080, and rs113414207) and four previously unreported variants (ss825679004, ss825679005, ss825679006, and ss825679007 (Table 1, *Supplementary Fig. 1, Supplementary Fig. 2*).

**Table 1:** Test of association with *ANK3* and *CACNA1C* variants with bipolar disorder

Gene	SNP	Location (hg19)	Position in gene	Change	European 1,000G MAF	MLP		Number of samples	Genotype counts	MAF	p-value^a^
*ANK3*	rs184389434	10: 62493837	Promoter	A/T	0	1.39	BP	1,469	0/15/1454	0.0051	0.41
							Control	1,031	0/12/1019	0.0058	
*ANK3*	ss825679002	10: 61788626	3′UTR	C/A	0	1.39	BP	1,467	0/36/1431	0.012	0.35
							Control	1,026	0/28/998	0.014	
*ANK3*	rs139972937	10: 61832711	Exon (N2643S)	A/G	0	1.39	BP	1,474	0/9/1465	0.0031	0.042
							Control	1,030	0/1/1029	0.0005	
*CACNA1C*	ss825679004	12: 2161934	Promoter	G/A	0	1.39	BP	1,467	0/5/1462	0.0017	0.57
							Control	1,031	0/4/1027	0.0019	
*CACNA1C*	ss825679005	12: 2694668	Splice site	G/A	0	1.39	BP	1,473	0/3/1470	0.0010	0.7
							Control	1,032	0/2/1030	0.0010	
*CACNA1C*	rs146482058	12: 2403077	Intron^b^^,^^e^	–/T	0	10.9	BP	1,486	3/134/1322	0.048	0.31
							Control	1,022	4/96/922	0.051	
*CACNA1C*	rs79398153	12: 2295156	Intron^b^^,^^e^	C/T	0.01	1.57	BP	1,498	3/88/1380	0.032	0.015
							Control	1,029	0/44/985	0.021	
*CACNA1C*	rs191953785	12: 2425097	Intron^b^^,^^e^	C/T	0	2.29	BP	1,475	0/25/1450	0.0085	0.34
							Control	1,020	0/20/1005	0.0098	
*CACNA1C*	rs112312080	12: 2354510	Intron^b^^,^^c^^,^^e^	C/T	0.001	2.02	BP	1,501	0/35/1439	0.012	0.30
							Control	1,028	0/20/1008	0.0097	
*CACNA1C*	rs113414207	12: 2292742-2292743	Intron^b^^,^^d^^,^^e^	–/C	0	2.15	BP	1,497	0/76/1394	0.026	0.19
							Control	1,026	0/44/982	0.021	
*CACNA1C*	ss825679006	12: 2329069	Intron^b^^,^^d^^,^^e^	G/T	0	2.15	BP	1,498	0/14/1457	0.0048	0.43
							Control	1,033	0/8/1024	0.0039	
*CACNA1C*	ss825679007	12: 2423175	Intron^b^^,^^c^^,^^d^^,^^e^	G/C	0	2.15	BP	1,502	0/4/1471	0.0014	0.34
							Control	1,026	0/1/1025	0.0010	

1,000G = 1,000 Genomes; *ANK3* = ankyrin 3; BP = bipolar disorder; *CACNA1C* = L-type calcium channel α1C subunit; MAF = minor allele frequency; SNP = single nucleotide polymorphism; UTR = untranslated region; European 1,000G MAF = MAF for European samples in the 1,000G Project 72; MLP = minus log_10_ of Fisher's exact p-value comparing UCL whole-genome sequence allele frequency to the European 1,000G MAF.

a p-significance value for a Fisher's exact test.

b H3K4Me1, H3K4Me3, and H3K27Ac marks.

c DNAse hypersensitivity cluster marks.

d Human mRNA.

e Conserved region. The distributions of genotype data for all SNPs were in Hardy–Weinberg equilibrium in the cases and controls.

The *ANK3* variant rs184389434 is located in the promoter region of the gene and was predicted to create a binding site for three new transcription factors [ETS-related gene (Erg-1), Ultrabithorax (Ubx) and Octamen-1 (Oct-1)]. rs139972937 causes a non-conservative amino acid change from asparagine to serine at position 2,643 (N2643S) in exon 34 of the alternative isoforms NM_020987.3 and CCDS7258.1. The N2643S amino acid substitution was predicted to be *benign* with a score of 0 (sensitivity 1, specificity 0) by PolyPhen-2 and *tolerated* with a score of 0.71 by SIFT. The previously unreported *ANK3* variant, ss825679002, was located in the 3′UTR of the gene and was found to be in a microRNA binding site. Bioinformatic analysis using targetscan and miRanda predicted no effect on microRNA binding.

One of the novel variants in *CACNA1C*, ss825679004, was in the promoter region of the gene. Alibaba 2.1 analysis of ss825679004 predicted it to disrupt the binding sites for three transcription factors [Activating Enhancer Binding Protein-2alpha (AP-2alph), NF-muE1, Specificity Protein-1 (Sp1)] and to create a new one for a different transcription factor [GC Factor (GCF)]. *CACNA1C* variant ss825679005 is the sixth base in the intron of the splice donor site for exon 17. This exon is present in all known isoforms of the gene and this variant might alter splicing efficiency.

The *CACNA1C* intron 3 variants rs146482058, rs79398153, rs191953785, rs112312080, rs113414207, ss825679006, and ss825679007 were present in the region of high LD between chr12:2,230,353 and chr12:2,559,413. Each variant was also located in an ENCODE marked region 65. All seven SNP regions are marked by H3 mono-methylation of lysine 4 (H3K4me1), H3 tri-methylation of lysine 4 (H3K4me3), and H3 acetylation of lysine 27 (H3K27ac), and active transcriptional enhancers with distinct chromatin signatures 68. Enrichment for H3K4me1 and H3K27ac at a genetic level distinguishes active enhancers from inactive or poised enhancers 69,70. The presence of H3K4me1- and H3K27ac-marked chromatin, with low levels of H3K4me3 and an absence of another histone marker, H3K27me3, represent putative human embryonic stem cell (hESC) enhancers and have been shown to localize proximally to genes that are expressed during development in hESCs and in epiblast cells 70. Additionally, rs112312080 and ss825679007 were found to be present on DNAse I hypersensitivity sites, as listed by the ENCODE project.

### Genotyping

Assays were designed for 13 SNPs which passed filtering tests for genotyping in the complete UCL BP case–control sample. Genotype data were generated for 12 of these variants and the genotype distributions for each SNP followed HWE in the case and control cohorts. The non-synonymous *ANK3* variant rs139972937 was found to be associated with BP (Fisher's exact test p = 0.042) (Table 1). Nine cases with BP and only one control were found to be heterozygous for this variant. None of the other *ANK3* variants were found to be associated with BP, as shown in Table 1.

Of the SNPs found in the *CACNA1C* intronic region, rs79398153 was found to be associated with BP (Fisher's exact test p = 0.015). We detected 88 heterozygote and three homozygote cases for this variant and 44 heterozygote controls. The excess of homozygotes may possibly represent a recessive effect, as only one homozygote would be expected under HWE, but this excess is not statistically significant 71. rs79398153 is located in an ENCODE-marked region for H3K4me1, H3K27ac, and H3Kme3. None of the other intronic CACNA1C SNPs were associated with BP. Imputation for rs79398153 in UCL1 using GWAS data showed that it was still significantly associated with BP (p = 0.022) 17. Burden analysis showed that, overall, there was no excess of the variants genotyped in cases versus controls for either *CACNA1C* or *ANK3*.

Haplotype and LD analysis at *ANK3* and *CACNA1C* was performed separately in Haploview 66 to examine the co-occurrence of the BP GWAS SNP alleles [*ANK3* rs10994285, rs2018783, rs10994336, rs10994397, rs10821792, rs1938526 14,26,32–34; *CACNA1C* rs1006737 14; rs4765913 14; rs476590 31; and rs4765914 20] with the BP risk variant alleles (rs139972937, allele G; rs79398153, allele T). For both sets of analyses, the BP risk variant alleles reported here were found to be associated with the GWAS SNP alleles that were more common in the control subjects. Thus, these variants could not be accounting for the GWAS signals. The results of the LD analysis are shown in Table 2.

**Table 2:** Pairwise linkage disequilibrium analysis between bipolar disorder-associated SNPs reported here and previous GWAS SNPs

Gene	Marker 1	Marker 2	D′	*r*^2^	LOD
*ANK3*	rs139972937	rs10994285	1	0	0.17
*ANK3*	rs139972937	rs2018783	1	0.002	0.51
*ANK3*	rs139972937	rs10994336	1	0	0.03
*ANK3*	rs139972937	rs10994397	1	0	0.03
*ANK3*	rs139972937	rs10821792	1	0	0.21
*ANK3*	rs139972937	rs1938526	1	0	0.26
*CACNA1C*	rs79398153	rs1006737	0.754	0.016	1.06
*CACNA1C*	rs79398153	rs1024582	0.729	0.014	0.87
*CACNA1C*	rs79398153	rs4765913	0.822	0.029	2.12
*CACNA1C*	rs79398153	rs4765914	0.829	0.032	2.31

*ANK3* = ankyrin; *CACNA1C* = L-type calcium channel α1C subunit; GWAS = genome-wide association studies; LOD = logarithm of the odds; SNPs = single nucleotide polymorphisms.

## Discussion

We have analysed genetic variation by WGS in two of the best-replicated bipolar susceptibility genes, *CACNA1C* and *ANK3*. This analysis has identified novel possible BP susceptibility variants in both of these genes. The *CACNA1C* intron 3 variant rs79398153 may impact *CACNA1C* gene expression by virtue of its presence in an ENCODE-marked transcriptional enhancer region.

The *ANK3* amino acid changing variant, rs139972937 (N2643S), was associated with BP in our sample (p = 0.042). This rare variant was also found in two of 1,119 cases and one of 1,078 controls and in a family containing seven subjects with BP, a father and six offspring, where only the father and two of the offspring possessed the variant 44. These conflicting findings are not uncommon in the genetics of complex diseases but this lack of support casts doubt on the true aetiological importance of this variant in BP.

It is of note that the allele frequencies of some of the variants selected for genotyping were found to be markedly different in our control samples compared to the frequencies in the European 1,000G Project. This underlies the importance of typing variants in matched control samples on the same platform rather than relying on publically available data such as the 1,000G Project in order to mitigate possible spurious association findings.

The variants we have found appear to be acting independently of the allelic and haplotypic associations found in the previous BP allelic association studies. Independent genetic replication and biological validation of intronic potential aetiological bp changes would support the argument for carrying out WGS as well as exome sequencing in BP. The biphasic nature of BP makes a compelling argument for the existence of genetically determined pathological switch mechanisms that may manifest themselves in the loss of control of gene expression. Findings such as ours may help to explain the ‘missing heritability’ in this common complex disorder. Further analyses in much larger samples are needed to find aetiological bp changes in the *ANK3* and *CACNA1C* genes that are carried by the main haplotypes showing strong association with BP. The outcome could be personalized treatment for BP, based on susceptibility genotypes.

## Supporting Information

**Figure S1.** The introns and exons of the different splice variants of the *ANK3* gene are shown, along with the genomic regions of the gene that were analysed for variant selection. The locations of the variants detected by sequencing are shown, as are the variants that were selected for genotyping in the full case control sample.

**Figure S2.** The introns and exons of the different splice variants of the *CACNA1C* gene are shown, along with the genomic regions of the gene that were analysed for variant selection. The locations of the variants detected by sequencing are shown, as are the variants that were selected for genotyping in the full case–control sample.

**Table S1.** Variants identified using the Knome VARIANTS software (Knome). VAF = variant allele(s) frequency.

**Table S2.** Allele-specific primers designed using Primer Picker (KBiosciences) for genotyping.

**Table S3.** Next Generation Sequencing Control information.

## References

[1] Merikangas KR, Jin R, He JP (2011). Prevalence and correlates of bipolar spectrum disorder in the world mental health survey initiative. Arch Gen Psychiatry.

[2] Bertelsen A (1987). A Danish twin study of manic-depressive disorders. Br J Psychiatry.

[3] Barnett JH, Smoller JW (2009). The genetics of bipolar disorder. Neurosci.

[4] Kendler KS, Pedersen NL, Farahmand BY, Persson PG (1996). The treated incidence of psychotic and affective illness in twins compared with population expectation: a study in the Swedish Twin and Psychiatric Registries. Psychol Med.

[5] Kieseppa T, Partonen T, Haukka J, Kaprio J, Lonnqvist J (2004). High concordance of bipolar I disorder in a nationwide sample of twins. Am J Psychiatry.

[6] McGuffin P, Rijsdijk F, Andrew M (2003). The heritability of bipolar affective disorder and the genetic relationship to unipolar depression. Arch Gen Psychiatry.

[7] Shih RA, Belmonte PL, Zandi PP (2004). A review of the evidence from family, twin and adoption studies for a genetic contribution to adult psychiatric disorders. Int Rev Psychiatry.

[8] Badner JA, Gershon ES (2002). Meta-analysis of whole-genome linkage scans of bipolar disorder and schizophrenia. Mol Psychiatry.

[9] Segurado R, Detera-Wadleigh SD, Levinson DF (2003). Genome scan meta-analysis of schizophrenia and bipolar disorder, part III: bipolar disorder. Am J Hum Genet.

[10] McQueen MB, Devlin B, Faraone SV (2005). Combined analysis from eleven linkage studies of bipolar disorder provides strong evidence of susceptibility loci on chromosomes 6q and 8q. Am J Hum Genet.

[11] Gurling H, Rifkin L, Horton KW, Katona C (1991). Genetic aspects of affective disorders. Biological Aspects of Affective Disorders.

[12] Spence MA, Flodman PL, Sadovnick AD (1995). Bipolar disorder: evidence for a major locus. Am J Med Genet.

[13] Craddock N, Khodel V, Van Eerdewegh P, Reich T (1995). Mathematical limits of multilocus models: the genetic transmission of bipolar disorder. Am J Hum Genet.

[14] Ferreira MA, O'Donovan MC, Meng YA (2008). Collaborative genome-wide association analysis supports a role for ANK3 and CACNA1C in bipolar disorder. Nat Genet.

[15] Chen DT, Jiang X, Akula N (2013). Genome-wide association study meta-analysis of European and Asian-ancestry samples identifies three novel loci associated with bipolar disorder. Mol Psychiatry.

[16] Wray NR, Pergadia ML, Blackwood DH (2012). Genome-wide association study of major depressive disorder: new results, meta-analysis, and lessons learned. Mol Psychiatry.

[17] Sklar P, Ripke S, Scott LJ (2011). Large-scale genome-wide association analysis of bipolar disorder identifies a new susceptibility locus near ODZ4. Nat Genet.

[18] Sklar P, Smoller JW, Fan J (2008). Whole-genome association study of bipolar disorder. Mol Psychiatry.

[19] Consortium WTCC (2007). Genome-wide association study of 14,000 cases of seven common diseases and 3,000 shared controls. Nature.

[20] Smoller JW, Craddock N, Kendler K (2013). Identification of risk loci with shared effects on five major psychiatric disorders: a genome-wide analysis. Lancet.

[21] Group PGCBDW (2011). Large-scale genome-wide association analysis of bipolar disorder identifies a new susceptibility locus near ODZ4. Nat Genet.

[22] Roussos P, Giakoumaki SG, Georgakopoulos A, Robakis NK, Bitsios P (2011). The CACNA1C and ANK3 risk alleles impact on affective personality traits and startle reactivity but not on cognition or gating in healthy males. Bipolar Disord.

[23] Jogia J, Ruberto G, Lelli-Chiesa G (2011). The impact of the CACNA1C gene polymorphism on frontolimbic function in bipolar disorder. Mol Psychiatry.

[24] Bhat S, Dao DT, Terrillion CE (2012). CACNA1C (Cav1.2) in the pathophysiology of psychiatric disease. Prog Neurobiol.

[25] Small SA, Schobel SA, Buxton RB, Witter MP, Barnes CA (2011). A pathophysiological framework of hippocampal dysfunction in ageing and disease. Nat Rev Neurosci.

[26] Tesli M, Koefoed P, Athanasiu L (2011). Association analysis of ANK3 gene variants in nordic bipolar disorder and schizophrenia case–control samples. Am J Med Genet B Neuropsychiatr Genet.

[27] Franke B, Vasquez AA, Veltman JA (2010). Genetic variation in CACNA1C, a gene associated with bipolar disorder, influences brainstem rather than gray matter volume in healthy individuals. Biol Psychiatry.

[28] Perrier E, Pompei F, Ruberto G (2011). Initial evidence for the role of CACNA1C on subcortical brain morphology in patients with bipolar disorder. Eur Psychiatry.

[29] Kempton MJ, Ruberto G, Vassos E (2009). Effects of the CACNA1C risk allele for bipolar disorder on cerebral gray matter volume in healthy individuals. Am J Psychiatry.

[30] Tesli M, Egeland R, Sønderby IE (2013). No evidence for association between bipolar disorder risk gene variants and brain structural phenotypes. J Affect Disord.

[31] Liu Y, Blackwood DH, Caesar S (2011). Meta-analysis of genome-wide association data of bipolar disorder and major depressive disorder. Mol Psychiatry.

[32] Schulze TG, Detera-Wadleigh SD, Akula N (2009). Two variants in Ankyrin 3 (ANK3) are independent genetic risk factors for bipolar disorder. Mol Psychiatry.

[33] Scott LJ, Muglia P, Kong XQ (2009). Genome-wide association and meta-analysis of bipolar disorder in individuals of European ancestry. Proc Natl Acad Sci U S A.

[34] Lett TA, Zai CC, Tiwari AK (2011). ANK3, CACNA1C and ZNF804A gene variants in bipolar disorders and psychosis subphenotype. World J Biol Psychiatry.

[35] Smith EN, Bloss CS, Badner JA (2009). Genome-wide association study of bipolar disorder in European American and African American individuals. Mol Psychiatry.

[36] Belmonte Mahon P, Pirooznia M, Goes FS (2011). Genome-wide association analysis of age at onset and psychotic symptoms in bipolar disorder. Am J Med Genet B Neuropsychiatr Genet.

[37] Green EK, Hamshere M, Forty L (2013). Replication of bipolar disorder susceptibility alleles and identification of two novel genome-wide significant associations in a new bipolar disorder case–control sample. Mol Psychiatry.

[38] Gella A, Segura M, Durany N (2011). Is Ankyrin a genetic risk factor for psychiatric phenotypes?. BMC Psychiatry.

[39] Kloiber S, Czamara D, Karbalai N (2012). ANK3 and CACNA1C–missing genetic link for bipolar disorder and major depressive disorder in two German case–control samples. J Psychiatr Res.

[40] Takata A, Kim SH, Ozaki N (2011). Association of ANK3 with bipolar disorder confirmed in East Asia. Am J Med Genet B Neuropsychiatr Genet.

[41] Lee MT, Chen CH, Lee CS (2011). Genome-wide association study of bipolar I disorder in the Han Chinese population. Mol Psychiatry.

[42] Gonzalez SD, Xu C, Ramirez ME (2013). Family-based association of an ANK3 haplotype with bipolar disorder in Latino populations. Transl Psychiatry.

[43] Dedman A, McQuillin A, Kandaswamy R (2012). Sequencing of the ANKYRIN 3 gene (ANK3) encoding ankyrin G in bipolar disorder reveals a non-conservative amino acid change in a short isoform of ankyrin G. Am J Med Genet B Neuropsychiatr Genet.

[44] Doyle GA, Lai AT, Chou AD (2012). Re-sequencing of ankyrin 3 exon 48 and case–control association analysis of rare variants in bipolar disorder type I. Bipolar Disord.

[45] Rueckert EH, Barker D, Ruderfer D (2013). Cis-acting regulation of brain-specific ANK3 gene expression by a genetic variant associated with bipolar disorder. Mol Psychiatry.

[46] Pandey A, Davis NA, White BC (2012). Epistasis network centrality analysis yields pathway replication across two GWAS cohorts for bipolar disorder. Transl Psychiatry.

[47] Judy JT, Seifuddin F, Pirooznia M (2013). Converging evidence for epistasis between ANK3 and potassium channel gene KCNQ2 in bipolar disorder. Front Genet.

[48] Linke J, Witt SH, King AV (2012). Genome-wide supported risk variant for bipolar disorder alters anatomical connectivity in the human brain. Neuroimage.

[49] Chaddock CA, Barker GJ, Marshall N (2009). White matter microstructural impairments and genetic liability to familial bipolar I disorder. Br J Psychiatry.

[50] Sussmann JE, Lymer GK, McKirdy J (2009). White matter abnormalities in bipolar disorder and schizophrenia detected using diffusion tensor magnetic resonance imaging. Bipolar Disord.

[51] McIntosh AM, Muñoz Maniega S, Lymer GK (2008). White matter tractography in bipolar disorder and schizophrenia. Biol Psychiatry.

[52] McIntosh AM, Job DE, Moorhead TW (2005). White matter density in patients with schizophrenia, bipolar disorder and their unaffected relatives. Biol Psychiatry.

[53] Lin F, Weng S, Xie B, Wu G, Lei H (2011). Abnormal frontal cortex white matter connections in bipolar disorder: a DTI tractography study. J Affect Disord.

[54] Sui J, Adali T, Pearlson GD, Calhoun VD (2009). An ICA-based method for the identification of optimal FMRI features and components using combined group-discriminative techniques. Neuroimage.

[55] Adida M, Jollant F, Clark L (2011). Trait-related decision-making impairment in the three phases of bipolar disorder. Biol Psychiatry.

[56] Bora E, Yucel M, Pantelis C (2009). Cognitive endophenotypes of bipolar disorder: a meta-analysis of neuropsychological deficits in euthymic patients and their first-degree relatives. J Affect Disord.

[57] Chandler RA, Wakeley J, Goodwin GM, Rogers RD (2009). Altered risk-aversion and risk-seeking behavior in bipolar disorder. Biol Psychiatry.

[58] McKirdy J, Sussmann JE, Hall J (2009). Set shifting and reversal learning in patients with bipolar disorder or schizophrenia. Psychol Med.

[59] McQuillin A, Rizig M, Gurling HM (2007). A microarray gene expression study of the molecular pharmacology of lithium carbonate on mouse brain mRNA to understand the neurobiology of mood stabilization and treatment of bipolar affective disorder. Pharmacogenet Genomics.

[60] Nanavati D, Austin DR, Catapano LA (2011). The effects of chronic treatment with mood stabilizers on the rat hippocampal post-synaptic density proteome. J Neurochem.

[61] Leussis MP, Berry-Scott EM, Saito M (2013). The ANK3 bipolar disorder gene regulates psychiatric-related behaviors that are modulated by lithium and stress. Biol Psychiatry.

[62] Spitzer RL, Endicott J, Robins E (1978). Research diagnostic criteria: rationale and reliability. Arch Gen Psychiatry.

[63] Spitzer R, Endicott J (1977). The Schedule for Affective Disorder and Schizophrenia, Lifetime Version.

[64] Stephens RM, Schneider TD (1992). Features of spliceosome evolution and function inferred from an analysis of the information at human splice sites. J Mol Biol.

[65] Consortium TEP (2004). The ENCODE (ENCyclopedia Of DNA Elements) Project. Science.

[66] Barrett JC, Fry B, Maller J, Daly MJ (2005). Haploview: analysis and visualization of LD and haplotype maps. Bioinformat.

[67] Jia P, Wang L, Fanous AH (2012). A bias-reducing pathway enrichment analysis of genome-wide association data confirmed association of the MHC region with schizophrenia. J Med Genet.

[68] Heintzman ND, Stuart RK, Hon G (2007). Distinct and predictive chromatin signatures of transcriptional promoters and enhancers in the human genome. Nat Genet.

[69] Creyghton MP, Cheng AW, Welstead GG (2010). Histone H3K27ac separates active from poised enhancers and predicts developmental state. Proc Natl Acad Sci U S A.

[70] Rada-Iglesias A, Bajpai R, Swigut T (2011). A unique chromatin signature uncovers early developmental enhancers in humans. Nature.

[71] Curtis D (2013). Consideration of plausible genetic architectures for schizophrenia and implications for analytic approaches in the era of next generation sequencing. Psychiatr Genet.

[72] Clarke L, Zheng-Bradley X, Smith R (2012). The 1000 Genomes Project: data management and community access. Nat Methods.

