# Supplementary material for: Analysis of ANK3 and CACNA1C variants identified in bipolar disorder whole genome sequence data
Source: Bipolar Disord. 2014 Apr 10;16(6):583–91. doi: 10.1111/bdi.12203 (PMC4227602; doi:10.1111/bdi.12203)
Supplement: Supplementary file 2 — Figure S2. The introns and exons of the different splice variants of the CACNA1C gene are shown, along with the genomic regions of the gene that were analysed for variant selection. The locations of the variants detected by sequencing are shown, as are the variants that were selected for genotyping in the full case–control sample. [file bdi0016-0583-SD2.doc]

Supplementary Figure 2


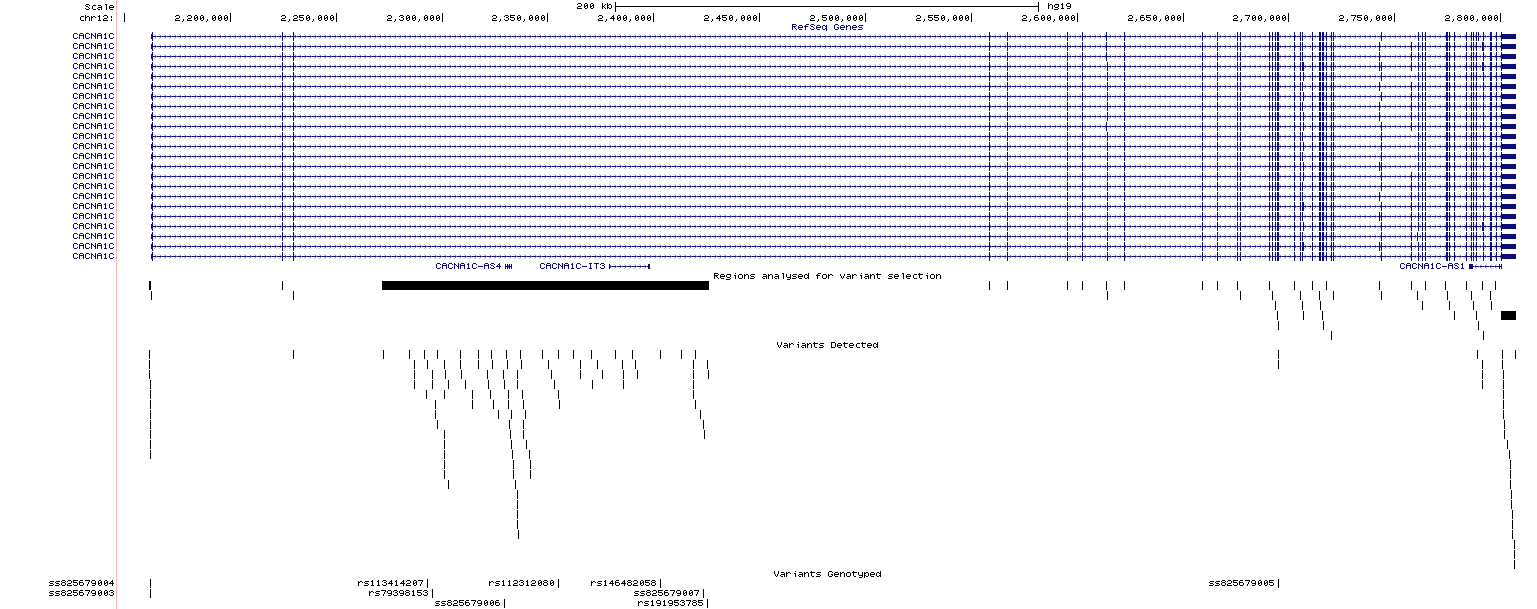


The introns and exons of the different splice variants of the CACNA1C gene are shown along with the genomic regions of the gene that were analysed for variant selection.

The locations of the variants detected by sequencing are shown as are the variants that were selected for genotyping in the full case control sample.
